# Microbial characterization based on multifractal analysis of metagenomes

**DOI:** 10.3389/fcimb.2023.1117421

**Published:** 2023-01-26

**Authors:** Xian-hua Xie, Yu-jie Huang, Guo-sheng Han, Zu-guo Yu, Yuan-lin Ma

**Affiliations:** ^1^ Key Laboratory of Jiangxi Province for Numerical Simulation and Emulation Techniques, Gannan Normal University, Ganzhoiu, China; ^2^ Key Laboratory of Intelligent Computing and Information Processing of Ministry of Education and Hunan Key Laboratory for Computation and Simulation in Science and Engineering, Xiangtan University, Xiangtan, China; ^3^ School of Economics, Zhengzhou University of Aeronautics, Zhengzhou, China

**Keywords:** diversity index, multifractal, metagenome, gut metagenome, chaos game representation (CGR)

## Abstract

**Introduction:**

The species diversity of microbiomes is a cutting-edge concept in metagenomic research. In this study, we propose a multifractal analysis for metagenomic research.

**Method and Results:**

Firstly, we visualized the chaotic game representation (CGR) of simulated metagenomes and real metagenomes. We find that metagenomes are visualized with self-similarity. Then we defined and calculated the multifractal dimension for the visualized plot of simulated and real metagenomes, respectively. By analyzing the Pearson correlation coefficients between the multifractal dimension and the traditional species diversity index, we obtain that the correlation coefficients between the multifractal dimension and the species richness index and Shannon diversity index reached the maximum value when q = 0, 1, and the correlation coefficient between the multifractal dimension and the Simpson diversity index reached the maximum value when q = 5. Finally, we apply our method to real metagenomes of the gut microbiota of 100 infants who are newborn and 4 and 12 months old. The results show that the multifractal dimensions of an infant's gut microbiomes can distinguish age differences.

**Conclusion and Discussion:**

There is self-similarity among the CGRs of WGS of metagenomes, and the multifractal spectrum is an important characteristic for metagenomes. The traditional diversity indicators can be unified under the framework of multifractal analysis. These results coincided with similar results in macrobial ecology. The multifractal spectrum of infants’ gut microbiomes are related to the development of the infants.

## Introduction

The study of species diversity in ecology has a long history (Kempton and Taylor, 1976; Hubalek, 2000). The diversity indices can be divided into two categories:α diversity index and β diversity index. All diversity indices referred to in this report are α diversity index which can be characterized by species richness, Shannon diversity index, and Simpson diversity index in macrobial (plants/animals). In the field of macrobial ecology, species richness increases with the increase of ecological area, and species–area relationship (SAR) can be formulated as *S*(*A*)=*cA*, where *A* is area, *S*(*A*) is the number of species in *A*, *c* and *z* are constants. SAR is a famous formula in ecological study (Borda-de-Água et al., 2002). On the basis of SAR, Harte and Kinzig pointed out that the formula indicates the self-similarity of species number and area (Harte and Kinzig, 1997). As a main feature of fractals, self-similarity can be described by


$$z_{q}=\underset{A\to +\infty }{\lim}\frac{1}{1-q}\cdot \frac{\ln\sum_{i=1}^{S\left(A\right)}p_{i}^{q}}{\ln\left(A\right)}\text{ and }z_{1}=\underset{A\to +\infty }{\lim}\frac{-\ln\sum_{i=1}^{S\left(A\right)}p_{i}\ln\left(p_{i}\right)}{\ln\left(A\right)}$$

When *q*<0, *z_q_* emphasizes the character of rare species; when *q*>0,*z_q_* emphasizes the common species. z_0_ implies the relationship between the logarithm of species richness [ln(*S*(*A*))] and the logarithm of the area [ln(*A*)]. *z*_1_ implies the relationship between the logarithm of the Shannon diversity (SHD) index and the logarithm of the area. *z*_2_ implies the relationship between the logarithm of the Simpson diversity (SID) index and the logarithm of the area.

In microbial diversity studies, it remains a challenge to identify bacterial strains in metagenome and microbiome samples by using computational analysis of short-read sequences (Kuleshov et al., 2016); hence, the main difference in diversity indices between macrobial and microbial is that the concept of “species” has been substituted by “OTUs”. The number of operation taxonomic units (OTUs) within a community is akin to species richness within macrobial systems (Stegen et al., 2016). Similar to macrobial ecology, species richness, Shannon diversity index, and Simpson diversity index were used to describe the species diversity of a microbial community (Leinster and Cobbold, 2012). However, there is still a lack of study to unify these diversity indicators into a single framework.

Fractal analysis has been applied in DNA sequence analysis for more than 30 years (Joel, 1990; Berthelsen et al., 1992). For example, chaos game representation (CGR) is a classical method (Joseph and Sasikumar, 2006), and it can map DNA sequences into a unit square as follows:


$$CGR_{\text{i}}=CGR_{i-1}+0.5*\left(P_{i}-CGR_{i-1}\right),P_{i}=P_{A},P_{C},P_{G}\;or\;P_{T},$$

Where *P_A_* =(0,0),*P_c_* =(0,1),*P_G_* =(1,0), and *P_T_* =(1,1) correspond to four nucleotides A, C, G, and T, respectively, *CGR*_0_=(0.5,0.5).

According to Karamichalis et al. (2016), CGRs also have been subjected to multifractal analysis (which measures the degree of self-similarity within the image). Based on the visualization of DNA sequence, its multifractal spectrum (Vélez et al., 2010; Moreno et al., 2011) can be defined as follows:


(1) $$D_{q}\left(\epsilon \right)=\{\begin{array}{c} \begin{array}{cc} \frac{\sum_{i}\left(\frac{M_{\text{i}}}{M_{0}}\right)\ln\left(\frac{M_{\text{i}}}{M_{0}}\right)}{\ln\left(\epsilon \right)} & q=1, \end{array}\\ \begin{array}{cc} \frac{1}{1-q}\frac{\ln\left(\sum_{i}{\left(\frac{M_{\text{i}}}{M_{0}}\right)}^{q}\right)}{\ln\left(\epsilon \right)} & q\neq 1. \end{array} \end{array}$$

where *ϵ* is the side length of grid, *M_i_* is the count of point in the *i*th grid, and *M_0_* is the summation of all *M_i_*. Furthermore, the multifractal dimensions of DNA sequence can be defined by $D\left(q\right)=\underset{\epsilon \to 0}{\lim}D_{q}\left(\epsilon \right)$ . In practical computation, the above-mentioned formula can be rewritten as follows:


(2) $$\ln\left(\sum_{\text{i}}M_{i}^{q}\right)=D_{q}\left(\epsilon \right)\left(q-1\right)\ln\left(\epsilon \right)+\left(q-1\right)\ln\left(M_{0}^{q}\right)$$

Then, *D*(*q*) can be calculated by linear fitting $M_{\text{i}}^{q}$ and ln(*ϵ*) (Vélez et al., 2010; Moreno et al., 2011).

Inspired by Karamichalis et al. (2016), the research group of Vélez studied the *Caenorhabditis elegans* genome (Vélez et al., 2010) and the human genome (Moreno et al., 2011) by multifractal formalism. Their results showed that the human (*Homo sapiens*) genome has stronger multifractality than that of *C. elegans* at the chromosome level. Similarly, Zhou et al. (2005) studied the discrimination problem of coding and non-coding DNA sequence. Their results suggest that coding and non-coding DNA sequence have different multifractal characteristics in the same genome. Pandit et al. (2012) studied the classification of HIV-1 by using multifractal dimensions of genomes. These results suggested that multifractal characteristics can measure the complexity of genes and genomes. Recently, Olyaee et al. used the CGR method to extract several valuable features from genomic sequences of SARS-CoV-2 (Olyaee et al., 2020). In 2021, Kania and Sarapata, 2021 studied the robustness of the chaos game representation to mutations and its application in an alignment-free method. On the basis of fractal scaling analysis, latterly, Meraz et al. (2022) characterized the organization of the SARS-CoV-2 genome sequence.

In order to further study the generalization of CGR, in 2019, Ge et al., 2019 generalized CGR to higher-dimensional spaces while maintaining its bijection, keeping such a method sufficiently representative and mathematically rigorous compared to previous attempts. In this frame, Dick and Green studied the proteome-wide protein prediction problem by chaos game representations and deep learning (Dick and Green, 2020). Ni et al. (2021) studied the gene sequence phylogenetic problem by frequency chaos game representation with perceptual image hashing.

For additive methods for genomic signatures of CGR, Karamichalis et al. (2016) reported their research results. They proposed the general concept of additive DNA signature of a set (collection) of DNA sequences. For example, the composite DNA signature combines information from DNA fragments and organellar, and the assembled DNA signature combines information from many short DNA subfragments (e.g., 100 base pairs) of a given DNA fragment. They concluded that such additive signatures could be used with raw unassembled next-generation sequencing (NGS) read data when high-quality sequencing data are not available.

Motivated by Karamichalis et al. (2016), in this study, we apply the fractal and multifractal methods to species diversity analysis of microbiomes. First, we visualize the simulated metagenomes and real metagenomes. Then, we compute the multifractal dimensions of simulated metagenomes and study the relationship between their multifractal dimensions and species diversity indices. Last, we compute multifractal dimensions of real metagenomes of 100 infants’ gut microbiomes when they are newborn, 4 months, and 12 months.

## Materials, methods, and results

### Metagenome datasets

The whole genomic sequences (WGS) (.fasta files) were downloaded from the NCBI database (ftp://ftp.ncbi.nlm.nih.gov/genomes/). The WGS for real metagenomes (.gz files) were downloaded from the NCBI SRA database (https://www.ncbi.nlm.nih.gov/sra).

**Dataset 1**: Simulated high-diversity metagenome set generated from the genomes of 10 distantly related major bacterial species used in Dubinkina et al. (2016). For each simulated metagenome, the number of reads is 10M and the read length is 1,000 bp. The high-diversity set includes 100 metagenomes generated from the genomes of 10 distantly related major bacterial species accounting for more than 90% of all reads in the Chinese group. The species used in dataset 1 are listed in **Table 1**. The abundances in dataset 1 are listed in **Supplementary Table S1**. In this simulation, the number of reads is 100 K and the read length is 1,000 bp.

**Table 1:** Species and accession numbers used in dataset 1.

Organism	Accession number
*Akkermansia muciniphila ATCC BAA-835*	NC_010655.1
*Alistipes shahii WAL 8301*	NC_021030.1
*Bifidobacterium adolescentis ATCC 15703*	NC_008618.1
*Bacteroides vulgatus ATCC 8482*	NC_009614.1
*Coprococcus* sp. *ART55/1*	FP929039.1
*Eubacterium eligens ATCC 27750*	NC_012778.1
*Faecalibacterium prausnitzii A2-165*	ACOP02000001.1
*Lachnospiraceae bacterium 1_4_56FAA*	NZ_GL945163.1
*Prevotella copri DSM 18205*	NZ_GG703878.1
*Ruminococcus champanellensis type strain 18P13T*	NC_021039.1

**Dataset 2**: Simulated low-diversity metagenome set generated from the genomes of 10 closely related major bacterial species used in Dubinkina et al. (2016). The species used in dataset 2 are listed in **Table 2**. The abundances in dataset 2 are listed in **Supplementary Table S2**. In this simulation, the number of reads is 100 K and the read length is 1000 bp.

**Table 2:** Species and accession numbers used in dataset 2.

Organism	Accession number
*Bacteroides caccae strain ATCC 43185*	NZ_CP022412.2
*Bacteroides dorei CL03T12C01*	NZ_CP011531.1
*Bacteroides ovatus strain ATCC 8483*	NZ_CP012938.1
*Bacteroides ovatus V975*	NZ_LT622246.1
*Bacteroides ovatus SD CMC 3*f	NZ_ADMO01000156.1
*Bacteroides stercoris ATCC 43183*	NZ_DS499677.1
*Bacteroides thetaiotaomicron VPI-5482*	NC_004663.1
*Bacteroides uniformis ATCC 8492*	NZ_DS362249.1
*Bacteroides vulgatus ATCC 8482*	NC_009614.1
*Bacteroides xylanisolvens CL03T12C04*	NZ_JH724294.1

**Dataset 3**: There are 400 WGS for real metagenomes of 100 infants’ and their mother’s gut microbiota. It includes 300 infants’ fecal metagenomes when they are newborn, 4 months, and 12 months, and 100 fecal metagenomes of their mothers. This dataset was used in Bäckhed et al. (2015) and the accession number is PRJEB6456. The study was approved by the Regional Ethical Review Board in Lund. Informed consent was obtained from all mothers.

### Visualization of metagenomes

Consider the alphabet Ω={*A*, *C*, *G*, T} and let *S*={*s*_1,_ *s*_2, ,_ *s_m_*} be a WGS metagenome dataset, we set *s_i_* =*s_i_*_1_ *s_i_*_2_ *s_i_*,*_ni_* as the *i*th reads in *S*, and *s*_*ik*_∈*Ω* is the *k*th nucleotide of reads *s_i_*. To represent a WGS dataset of metagenome in the form of a CGR plot, a unit square was used, whose four vertices were labeled as *A*=(0,0), *C*=(0,1), *G*=(1,0), and *T*=(1,1). For a given metagenome dataset *S*={ s_1_,*s*_2_,⋯,*s*_*m*_ } , which includes *m* reads, the *k*th nucleotide of reads corresponds to *CGR*_ik_=*CGR*_i,k-1_+0.5^*^(*P_ik_*-CGR*_i,k_*_-1_,*P*_i_=*P_A_*,*P_C_*,*P_G_*, or *P_T_*, *i*=1,2,…,*m*, where *P_A_* =(0,0), *P_C_* =(0,1), *P_G_* =(1,0), and *P_T_* =(1,1) correspond to the four nucleotides A, C, G, and T, respectively,*CGR_i_*_0_=(0.5,0.5). In order to avoid “large number annihilating small number”, we discarded the first 10 points of each read.

**Figure 1:**
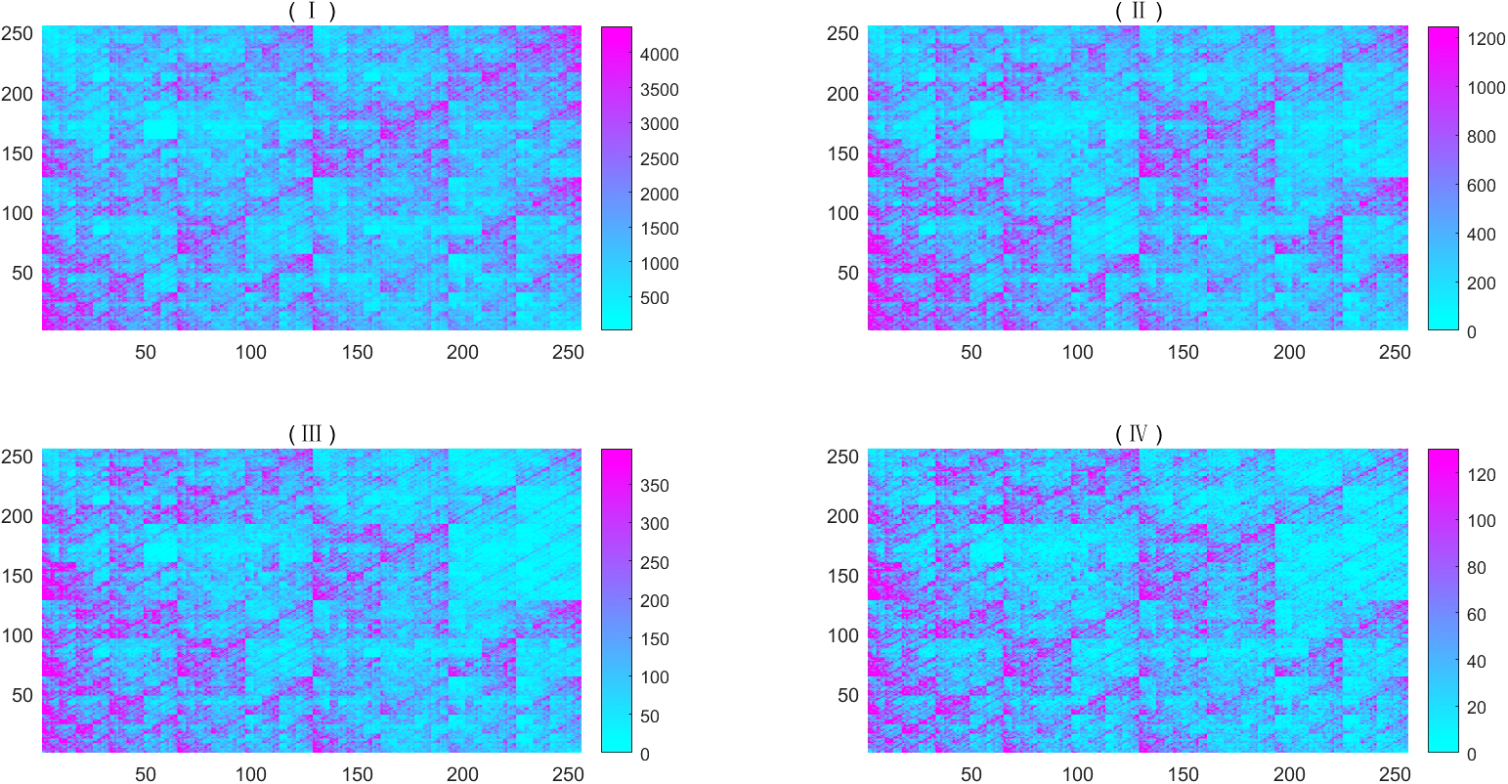
Heat map of simulated metagenome of dataset 2, the abundance are 0.016928832, 0.30559462, 0.120814049, 0, 0.07959993, 0.00894306, 0.03682631, 0.37768779, 0.00570905, and 0.04789635. The dissolution of (I) is 256×256; (II) is an image magnified by a factor of 2 from the upper left part of (I); (III) is an image magnified by a factor of 2 from the upper left part of (II); and (IV) is an image magnified by a factor of 2 from the upper left part of (III). For better visibility, we regarded the number exceeding the threshold as the threshold, whose values are taken three times the mean value.

Based on the plotting point above, we split the unit square into 256 × 256, 512 × 512, 1,024 × 1,024, and 2,048 × 2,048 small squares in turn and then we counted the number of points in every small square. **Figure 1** is an example of dataset 2.

### Fractal and multifractal spectrum of metagenome

From **Figure 1**, we found that all CGRs seem to be self-similar. Thus, we intended to study their fractal and multifractal properties. On the basis of visualization of metagenome sequence, one can define its multifractal spectrum by Eq. (1).

**Figure 2:**
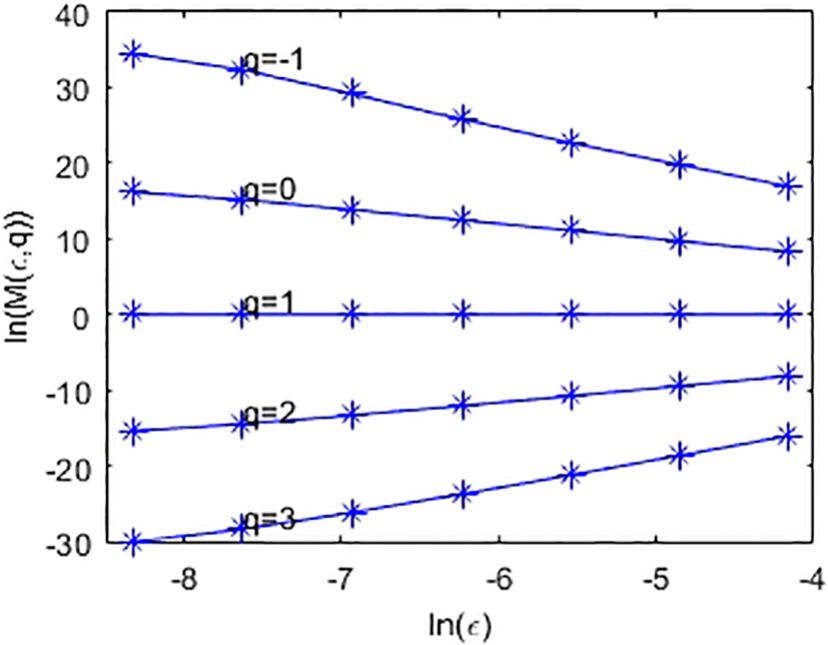
Linear fit of ln(M(*ϵ*,*q*)) and ln(*ϵ*), where *ϵ* is set to 2^-6^,2^-7^,2^-8^,2^-10^,2^-11^,2^-12^, and 2^-13^, respectively.

Furthermore, one can define multifractal dimension by $D\left(q\right)=\underset{\epsilon \to 0}{\lim}D_{q}\left(\epsilon \right)$ . In practical computation, one can compute *D*(q) by linear fitting between ln(M(*ϵ*,q)) and ln(*ϵ*) according to Eq. (2). **Figure 2** shows the linear fit between $\ln\left(\sum_{\text{i}}M_{\text{i}}^{q}\right)$ (i.e., ln(M(*ϵ*,q))) and ln(*ϵ*) of the simulated metagenome.

**Figure 3:**
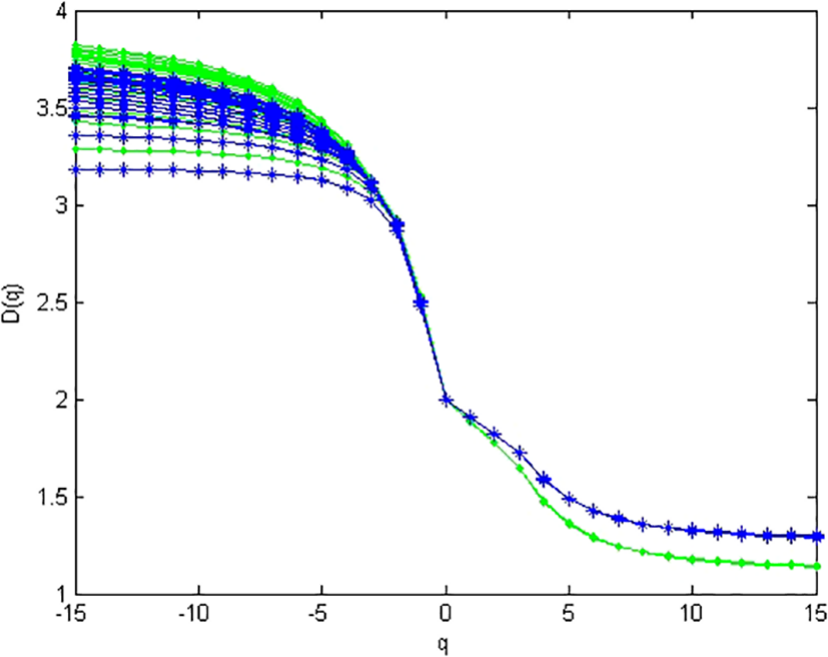
Multifractal dimensions of simulated genome. Green asterisks represented the D(q) of samples simulated from high-diversity communities, and blue dots represented the D(q) of samples simulated from low-diversity communities. For each sample from the same community, the abundances are given in **Table S1** (the last line in the table).

In metagenomic research, for a given community, a WGS dataset of metagenome is actually a collection of simple random-sampling reads from the given community (i.e., the abundance values of bacteria is fixed). In this experiment, we simulated 100 metagenomes from a given abundance of 10 bacteria. **Figure 3** demonstrates the multifractal dimensions of 100 simulated metagenomes (i.e., 100 simple random samplings) from dataset 1 and 100 simulated metagenomes from dataset 2. In a modest PC [Intel(R) Core(TM) i7-9750H CPU @ 2.60GHz, 8 GB RAM], the present method only needs 6 h 54 m 43 s for computing the multifractal dimensions of 100 simulated metagenomes from dataset 1 and 6 h 50 m 42 s for computing the multifractal dimensions of 100 simulated metagenomes from dataset2.

From **Figure 3**, we can find that multifractal dimension curves of different simulated metagenomes from the same abundance are unstable when *q* < 0, and they are stable when *q* ≥ 0. Hence, we only consider *D*(*q*) for *q* ≥ 0 in the multifractal spectrum of metagenome.

### The relationship between multifractal spectrum and microbial diversity indices of metagenomes

In order to study the relationship between the multifractal spectrum and diversity indices of metagenomes, we simulated 100 metagenomes whose abundance is known, and then their species richness index, Shannon diversity index, Simpson diversity index, and multifractal dimensions are calculated. Based on these results, the Pearson correlation coefficients are calculated according to varying *q*.

**Figure 4:**
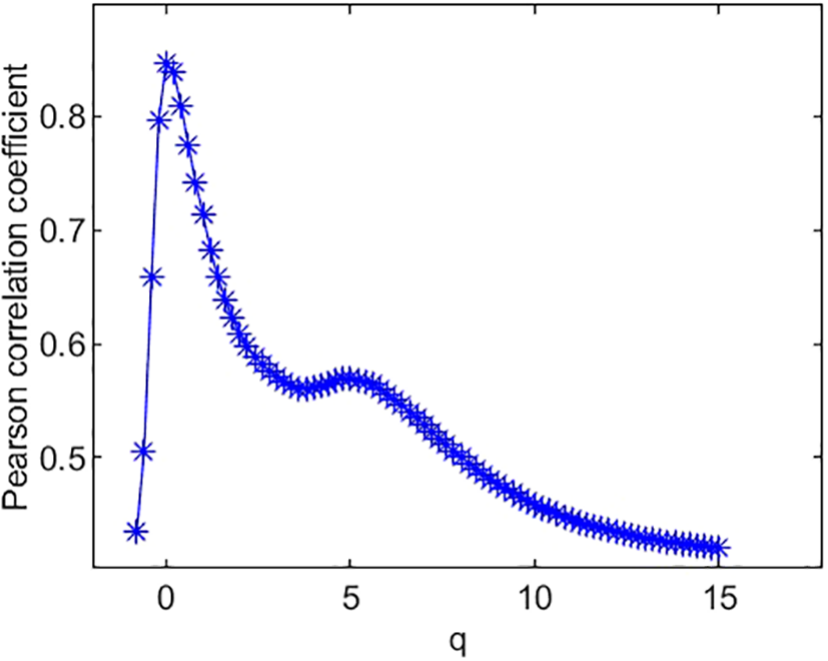
Pearson correlation coefficient of species richness and multifractal dimension *D*(*q*).

**Figure 5:**
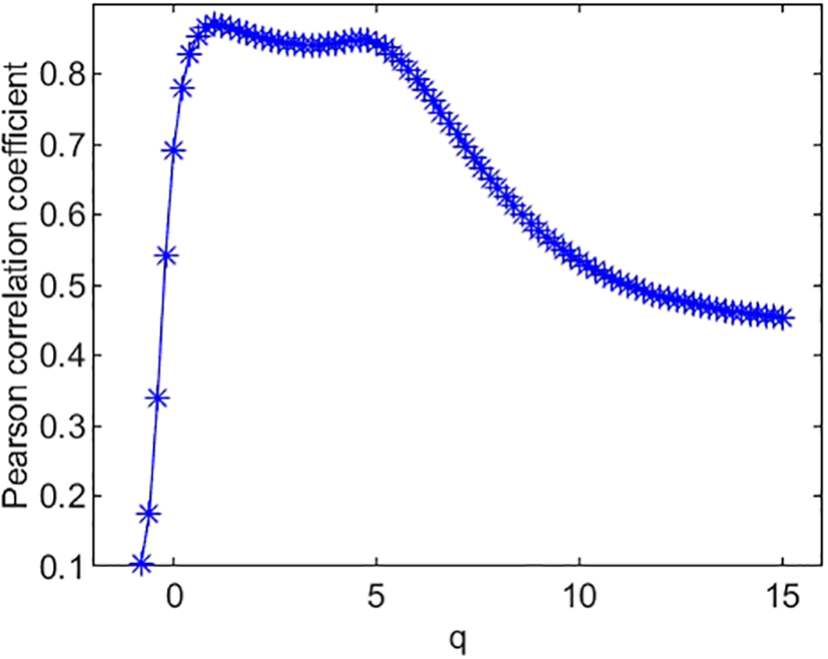
Pearson correlation coefficient of species’ Shannon diversity index and multifractal dimension *D*(*q*).

**Figure 6:**
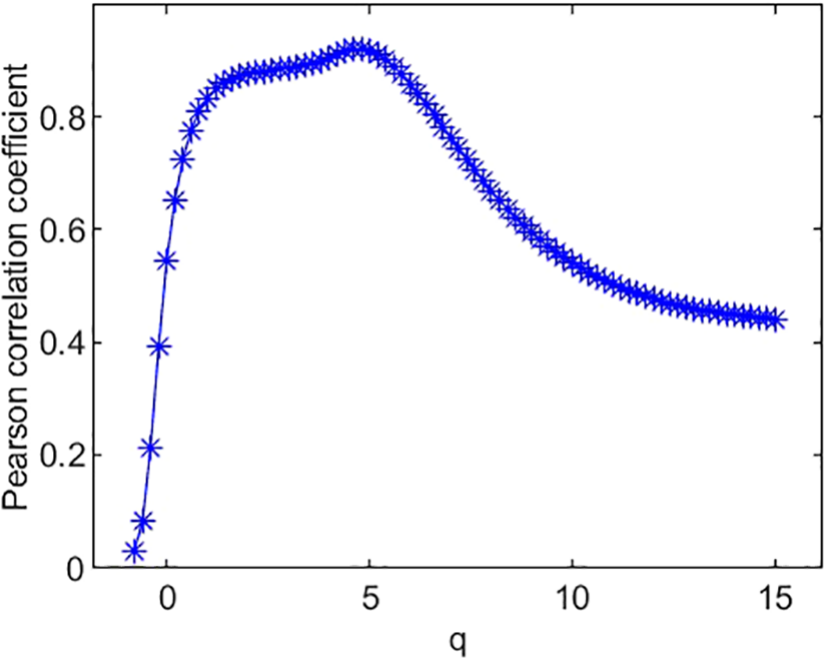
Pearson correlation coefficient of species’ Simpson diversity index and multifractal dimension *D*(*q*).

The Pearson correlation coefficients between species richness diversity indices and multifractal dimension are plotted in **Figure 4**. The plot suggests that the Pearson correlation coefficient between species richness indices and multifractal dimensions reach its maximum (0.85) at *q* = 0. Similarly, the Pearson correlation coefficients (**Figure 5**) between species Shannon diversity indices and multifractal dimensions reach its maximum (0.88) at *q* = 1. The Pearson correlation coefficient (**Figure 6**) between species Simpson diversity indices and multifractal dimensions reaches 0.87 at *q* = 2, while the Pearson correlation coefficients between species Simpson diversity indices and multifractal dimensions reach their maximum (0.89) at *q* = 5.

### Application of multifractal dimension in metagenomes to infant’s gut microbiome

In order to apply the multifractal analysis to real metagenomes, we selected 100 infants’ fecal WGS datasets of 300 metagenomes [there are three samples, including 12 months (12 M), 4 months (4 M), and newborn (baby) for each infant] and 100 corresponding gut metagenomes of their mothers to mine potential information of its multifractal dimensions.

**Figure 7:**
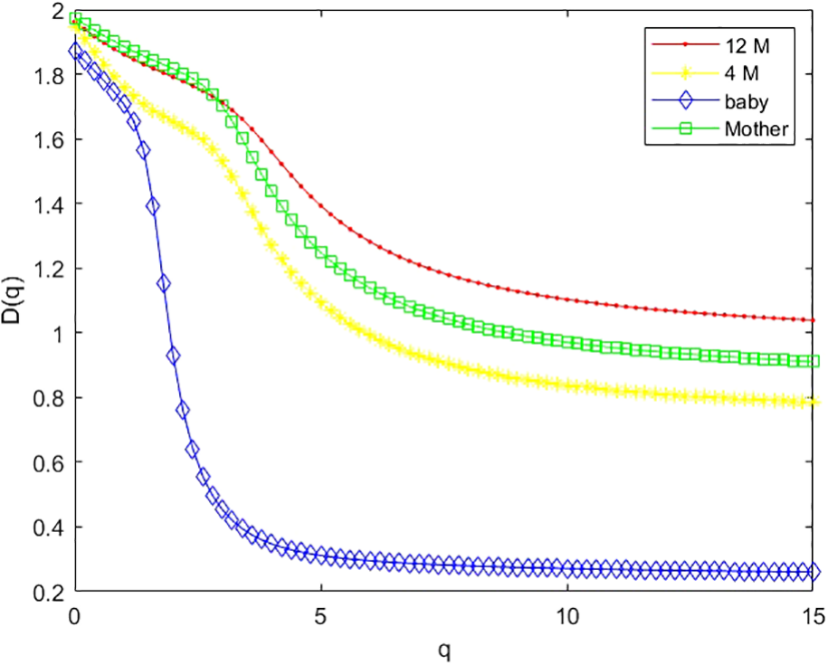
Multifractal dimension of the gut microbiome of the infant when he/she is 12 months old (12 M), 4 months old (4 M), and a newborn baby (baby), and her mother (M).

As an example, we plotted multifractal dimensions of a selected gut microbiome of a baby in **Figure 7**. The plot demonstrates the multifractal dimensions of gut microbiomes of an infant and his/her mother when he/she is a newborn (baby) and 4 months and 12 months old. Our method consumed 23 h 5 m 46 s for multi-fractal dimensions of the 400 metagenomes. **Figure 9** suggests that the *D*(0) (fractal dimension),*D*(1) (information dimension), and *D*(2) (correlation dimension) are increasing with growth. In other words, their gut microbial diversity is developing with growth. In order to study the generality of this property, we calculated the mean value and standard deviation of 100 multifractal dimensions of infants at 12 months and 4 months, when they were a baby, and their mothers, respectively.

**Figure 8:**
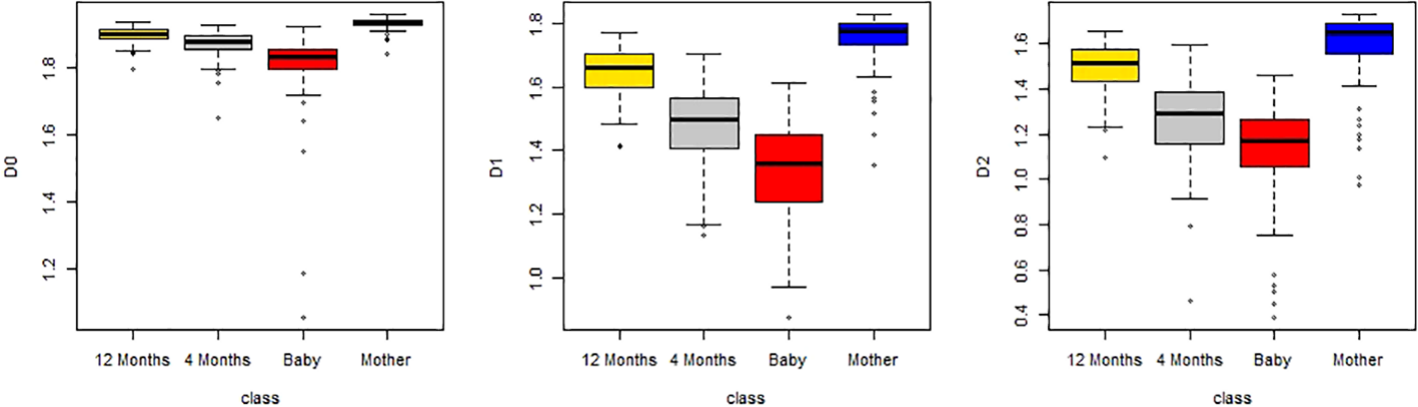
Boxplots of D(0), D(1), and D(2) of multifractal dimension of gut microbiomes of infants when they are 12 months old, 4 months old, and a baby, and that of the mother.

From **Figure 8**, we also found that *D*(0), *D*(1), and *D*(2) are increasing with growth in total. In order to obtain the statistical significance of these results, we tested their statistical significance, the results of which are shown in **Table 3**. From **Table 3**, we conclude that *D*(0), *D*(1), and *D*(2) are increasing with the growth in statistical significance.

**Table 3:** *p*-values of the one-sided *t*-test of dataset 3 (alternative hypothesis: less).

	Baby *vs*. 4 months	4 months *vs*. 12 months	12 months *vs* mother
D(0)	7.493e-07	5.548e-10	2.2e-16
D(1)	3.555e-11	2.2e-16	2.2e-16
D(2)	1.655e-06	2.2e-16	1.713e-06

**Figure 9:**
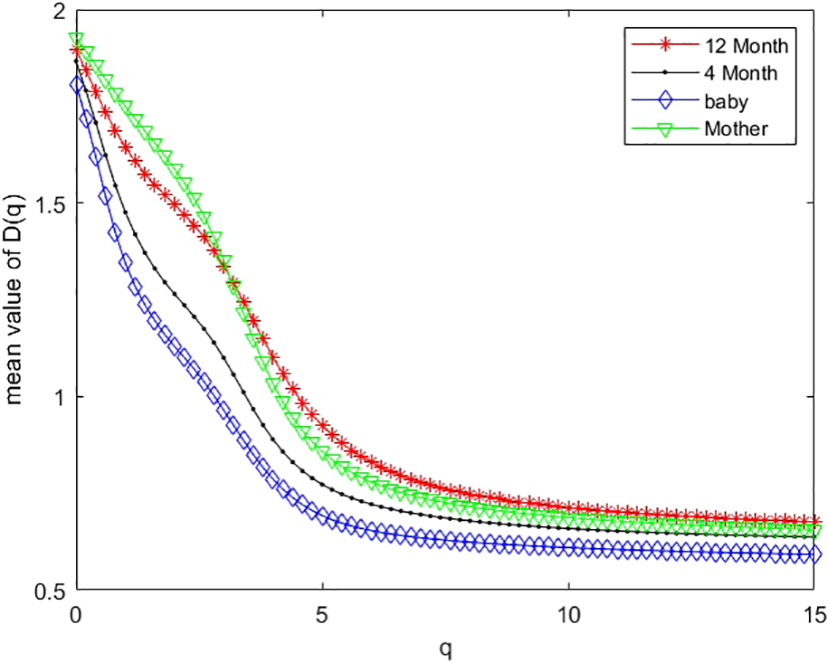
Mean values of multifractal dimension of 100 infants’ gut microbiomes when they are 12 months old (12 M), 4 months old (4 M), and a newborn baby (baby), and that of their mother (M).

For dataset 3, there are 100 infants’ gut microbiomes. We grouped each infant gut micobiome as one group, and there are 100 groups of gut microbiomes. For each group, we calculated the difference between 12 months and 4 months, 12 months and newborn, and 4 months and newborn, respectively. In order to observe the overall characteristics of these multifractal dimensions, we plotted the mean value of 100 multifractal dimensions of gut microbiomes in **Figure 9**. From **Figure 9**, we can draw similar conclusions to the above.

In order to evaluate the discriminating power of gut microbiomes’ multifractal dimensions in ages of infants, we used multifractal dimensions *D*(*q*) (*q* from to 15 with step 0.2) of infants at 12 months and 4 months, when they were a baby, and the mothers’ gut microbiomes to discriminate by linear discriminant analysis (LDA) in R (Xu et al., 2021). It consumed only 87 s to differentiate. **Table 4** demonstrates the back discriminant results of infants at 12 months and 4 months, when they were a baby, and mothers by LDA. From **Table 4**, we know that the accurate rate of the mothers’ gut microbiomes (90 out of 100) is best discriminated by multifractal dimensions. We also apply LDA with leave-one-out cross-validation to the dataset; the accuracy rate is 74.79%.

**Table 4:** Table of back discriminating results of 400 metagenomes.

	Mother	12 months	4 months	Baby
Mother	90	7	3	0
12 months	9	81	9	1
4 months	0	13	72	15
Baby	1	3	20	76

## Discussion and conclusions

In this study, we studied metagenomes by multifractal analysis. From the results above, we obtained the following conclusions:

(i) From the CGR visualization of metagenomes (**Figure 1**), we find that there exists statistical self-similarity in these CGR visualizations of metagenomes. From **Figure 2**, we concluded that there is linearity between $\ln\left(\sum_{\text{i}}M_{\text{i}}^{q}\right)$ (i.e., ln(M(*ϵ*,q))) and ln(*ϵ*) for simulated WGS metagenomes. **Figure 3** demonstrates 100 simulated WGS metagenome samples from two given abundance, suggesting that the *D*(*q*) of metagenomes is stable when *q*≥0 and unstable when *q*<0 [as we know, when *q*<0, *D*(*q*) emphasizes the rare species (k-mers); in nature, reads of metagenome were sampled from microbial genomes, and the copy numbers of rare k-mers are unstable, so that *D*(*q*) was unstable]. These results guide us to study multifractal dimensions of metagenomes only for *q≥*0 in the following study. These results show that there is a multifractal character in CGRs of WGS of metagenomes.

(ii) From **Figure 4**, we can see that the Pearson correlation coefficients of species richness indices and *D*(*q*) reach their maximums when *q*=0. Similarly, we can find that the Pearson correlation coefficients of Shannon diversity indices and *D*(*q*) reach their maximums when *q*=1 from **Figure 5**, and that the Pearson correlation coefficients of Simpson diversity indices and *D*(*q*) approach their maximums when *q*=2 (the maximums are valued at *q*=5) from **Figure 6**. These results roughly coincide with the results of macrobial ecology in [4]. On the whole, the scatter plot of Shannon diversity indices and the corresponding *D*(1) demonstrated in **Figure 5** show that *D*(1) is increasing with the increase of Shannon diversity indices of metagenomes. **Figure 6** shows that *D*(2) is increasing with the increase of Simpson diversity indices of metagenomes. These results show that there are linearly correlated relationships between multifractal dimensions and traditional diversity indices. They also suggest that multifractal dimensions can reflect the microbial diversity in metagenomic research and the traditional diversities can be unified by the frame of multifractal analysis.

(iii) In research on real metagenomes, the multifractal dimensions of the gut microbiome of one mother and her baby are demonstrated in **Figure 7**; this plot shows that the multifractal dimensions of gut microbiome of baby are increasing with the infants (newborn, 4 months, and 12 months). The boxplot of **Figures 8**, **9** show that this law holds on the whole for babies on average. The back discriminant results of multifractal dimensions of gut microbiomes of infants demonstrated in **Table 3** show that the infants’ age can be discriminated by their multifractal spectrum of CGR visualization of gut microbiomes in total. Specifically, newborn results are the best. The gut microbiomes of a 4-month-old baby can be confused more easily. For leave-one-out cross-validation, the accurate rate reached 74.97%, suggesting that the multifractal spectrum of gut microbiomes for infants can discriminate their ages powerfully.

In conclusion, there is self-similarity among the CGRs of WGS of metagenomes, and the multifractal spectrum is an important characteristic for metagenomes. The multifractal spectrum of metagenomes is related to species diversity and the development of gut microbiomes of infants.

In our study, the advantages were that the algorithm does not need alignment and that it required less computing resources than aligned methods. The disadvantage was that the algorithm cannot obtain a detailed composition and species abundance from metagenomes.

## Data availability statement

The datasets presented in this study can be found in online repositories. The names of the repository/repositories and accession number(s) can be found in the article/**Supplementary Material**.

## Ethics statement

The studies involving human participants were reviewed and approved by the Regional Ethical Review. Written informed consent to participate in this study was provided by the participants’ legal guardian/next of kin.

## Author contributions

Conceptualization, X-HX and Y-LM. Methodology, X-HX and Z-GY. Software, X-HX and Y-LM. Validation, X-HX, Y-LM, Z-GY and G-SH. Formal analysis, X-HX and Y-JH. Resources, X-HX. Data curation, X-HX. Writing—original draft preparation, X-HX and Z-GY. Writing—review and editing, X-HX and Z-GY. Visualization, X-HX. Supervision, Z-GY. All authors contributed to the article and approved the submitted version.

## Supplementary material

Supplementary Table 1 The abundances in dataset 1

Supplementary Table 2 The abundances in dataset 2.
